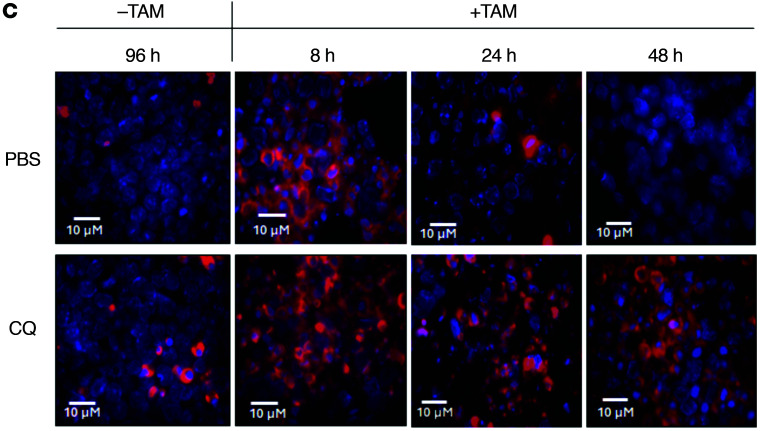# Corrigendum to Autophagy inhibition enhances therapy-induced apoptosis in a Myc-induced model of lymphoma

**DOI:** 10.1172/JCI195984

**Published:** 2025-07-15

**Authors:** Ravi K. Amaravadi, Duonan Yu, Julian J. Lum, Thi Bui, Maria A. Christophorou, Gerard I. Evan, Andrei Thomas-Tikhonenko, Craig B. Thompson

Original citation: *J Clin Invest*. 2007;117(2):326–336. https://doi.org/10.1172/JCI28833

Citation for this corrigendum: *J Clin Invest*. 2025;135(14):e195984. https://doi.org/10.1172/JCI195984

The authors recently became aware that in Figure 3C of the original article, the 8 h +TAM/CQ image was inadvertently duplicated as the 8 h +TAM/PBS panel. The correct panel, provided from the original source data, is shown below. The authors have stated that the correction does not affect the conclusions of the work.

The authors regret this error.

## Figures and Tables

**Figure 3C:**